# Genetic Parameter Estimates for Teat and Mammary Traits in Commercial Sows

**DOI:** 10.3390/ani13152400

**Published:** 2023-07-25

**Authors:** Audrey L. Earnhardt-San, Kent A. Gray, Mark T. Knauer

**Affiliations:** 1 Department of Animal Science, North Carolina State University, Raleigh, NC 27695, USA; alearnhardt@tamu.edu; 2 Smithfield Premium Genetics, Rose Hill, NC 28458, USA; kgray@smithfield.com

**Keywords:** functional teat, heritability, lactation, piglet survival, sow

## Abstract

**Simple Summary:**

The use of genetic selection in livestock has allowed farmers to enhance the sustainability of their operations by producing animals that require fewer inputs relative to animal outputs. In swine, geneticists have selected for sows that farrow larger litters, which has reduced the feed required per piglet produced. Yet challenges remain with piglet survival from birth to weaning. Utilizing genetic selection to increase the sow teat number may be a strategy to enhance piglet nutrient access and therefore piglet survival. More publicly available data are needed to confirm associations between sow teat traits and piglet weaning traits. Hence, the current study explores the genetic relationships among sow teat traits at farrowing with the number of piglets at weaning and identifies strategies to enhance piglet survival and sustainable pig production. The results suggest geneticists should incorporate the number of functional teats a sow has at farrowing into genetic selection programs to improve piglet survival and animal well-being.

**Abstract:**

The objective was to evaluate the genetics of sow teat and mammary traits at farrowing and at weaning. Data were recorded on 3099 Landrace × Large White F1 sows. Underline traits included the total teat number (TT), the functional teat number (FT), the non-functional teat number (NFT), the damaged teat number (DT), and the number of functional mammary glands (FMG). Variance components were estimated using AIREMLF90. Means for TT, FT, and NFT at farrowing were 14.93, 13.90, and 1.03, respectively. Heritability estimates for TT, FT, and NFT ranged from 0.18 to 0.37, 0.16 to 0.28, and 0.14 to 0.18, respectively. Estimates of heritability for DT and FMG at weaning were 0.03 and 0.06, respectively. Estimated genetic correlations between FT with TT and NFT were 0.68 to 0.78 and −0.19 to −0.57, respectively. Genetic correlation estimates between TT, FT, and NFT with the number weaned were 0.25, 0.50, and −0.38, respectively. An increase of one TT and FT enhanced (*p* < 0.05) the number weaned by 0.14 to 0.16 and 0.18 to 0.27 piglets, respectively. The results suggest that genetically increasing the number of functional teats on a sow at farrowing would improve the number of piglets at weaning.

## 1. Introduction

Enhancing piglet survival is dependent on teat accessibility, teat function, and teat number. Historic genetic selection of swine for increased litter sizes [1] and improved finishing traits (i.e., growth rate and carcass lean content) have perhaps inadvertently increased selection for other undesirable traits [2]. These undesirable traits include reduced accessibility to functional teats, which is unfavorably associated with piglet survival [3]. Reduced teat functionality and accessibility increases the risk of newborn piglets failing to intake sufficient amounts of colostrum. Both teat functionality and colostrum intake are rudimentary causes for decreased piglet weaning weights and the majority of early postpartum piglet deaths [4]. Long-term negative connotations, such as increased sibling competition, will continue with genetic selection for increased litter sizes. Thus, including underline traits in the breeding objective may help alleviate these negative consequences [2].

Understanding the genetics of underline traits in swine and including these traits in genetic selection will help enhance teat accessibility and sow maternal ability [5]. Knowledge of the genetics behind the functional teat number is of fundamental importance to facilitate teat availability and improve piglet survival and performance. Commonly, studies relating the sow teat number to subsequent reproduction evaluate teat count before gilts are mated [5,6,7,8,9] and generally not close to parturition. Therefore, studies evaluating sow teat count near lactation are warranted. Hence, the objective of the current study was to estimate variance components among sow teat and mammary traits in commercial sows.

## 2. Materials and Methods

### 2.1. Animals

Data (n = 3099 sows) from Landrace × Large White F1 females (Smithfield Premium Genetics, Rose Hill, NC, USA) within seven sow farms located in eastern North Carolina were used. In gestation, sows were housed in environmentally controlled buildings on concrete slatted floors, given ad libitum access to water, and fed based on sow body condition [10]. Prior to farrowing, sows were moved to individual lactation stalls where they were offered water and feed ad libitum. The lactation diet met or exceeded nutritional requirements [11]. Sows were housed in farrowing barns with a range of 10 to 30 farrowing stalls per room. Piglets were cross-fostered to balance the number of piglets nursing each sow, regardless of the functional teat number. While minimizing the number of piglet movements was encouraged, the largest piglets within a litter were transferred to smaller litters when cross-fostering was carried out. All cross-fostering was performed within 48 h of farrowing.

### 2.2. Underline Traits

**Figure 1:**
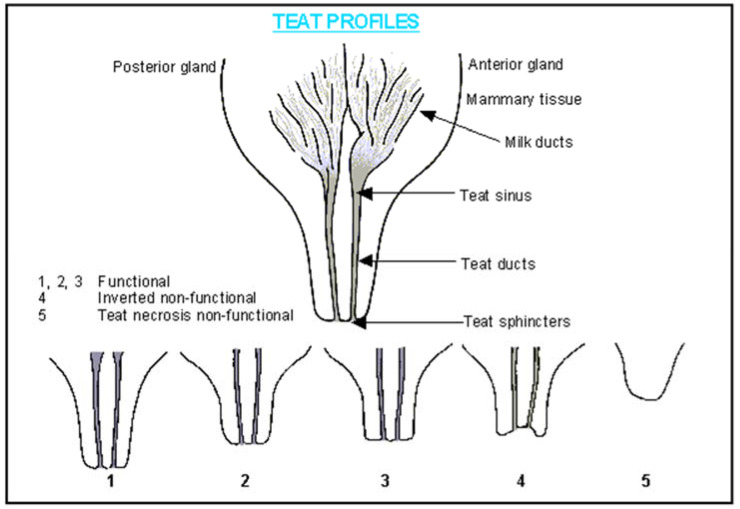
Teat classification system [12] used to differentiate between functional and non-functional teats.

Teat traits observed on all sows prior to farrowing and at weaning included the total teat number, the functional teat number, and the non-functional teat number. Trained technicians evaluated all teat traits. At weaning, the damaged teat number and functional mammary glands were also assessed. Teats were classified according to the scoring system (Figure 1) proposed by [12] and descriptions from the article “Genetics of teat number in swine” [13]. Functional teats were elongated teats that had a well-developed and predominant sphincter and non-functional teats were inverted (turned inward to form a crater) or supernumerary (small and shorter in size compared with normal teats) [13]. Damaged teats were teats injured during lactation by either piglets or the environment. At weaning, mammary glands were classified as functional if they appeared swollen with milk and had not entered mammary involution. The total teat number was calculated from the sum of the functional teat number, the non-functional teat number, and the damaged teat number.

### 2.3. Data Collection

Data collection occurred biweekly across the sow farms. Sows within five days of farrowing or within three days of weaning were assessed. Hence, a proportion of sows were sampled at both farrowing and weaning. Data were collected during feeding to ensure sows were standing while observed as suggested by [14]. When a sow is lying down there is an increased chance that the visibility of certain teats will be impaired, thus negatively influencing the accuracy of teat classification. Sow farrow date, parity, and the number of piglets born alive were recorded. The number of piglets weaned for every sow sampled was recorded and obtained from the Smithfield Premium Genetics database.

### 2.4. Data Editing

Data were edited using Mac Terminal v. 2.8.2 and RStudio v. 1.1.463. Data from 3401 sows were available for analysis. Records in which sows were not sampled within five days of farrowing or within three days prior to weaning were excluded. After editing, data from 3099 sows were retained for analysis. The numbers of records from sows at farrowing and at weaning were 1840 and 1259, respectively. Across all sows sampled, there were 300 unique sires, with 276 unique sires for sows sampled at farrowing and 235 unique sires for sows sampled at weaning.

### 2.5. Statistical Analysis

Descriptive statistics for each trait were calculated using RStudio v. 1.1.463. The genetic analyses utilized restricted maximum likelihood (REML) statistical models. Univariate and bivariate models were performed to estimate variance components for each trait: total teat number, functional teat number, non-functional teat number, damaged teat number, number of functional mammary glands, and number of piglets weaned. Pedigrees for sows sampled were provided by the PigKnows (Greeley, CO, USA) database to be utilized in the data analysis for variance component estimation. Pedigrees included five generations and comprised both the dam and the sire of the parents of the sow sampled. The RENUMF90 v. 1.122 program [15] was used to process the data by performing comprehensive pedigree checks and creating the parameter file for AIREMLF90 v. 1.135 [15]. The AIREMLF90 v. 1.135 program was applied to determine relationships between the observed traits. Models included fixed effects of sow parity, farm, underline evaluator, and production phase. Sow was included as a random effect. Common litter and sire effects were excluded from model analysis due to resulting variance estimates being negligible.

Heritability was calculated from the estimated variance component models using the following equation,

$$h^{2}=\frac{a}{\left(a+e\right)},$$

where $h^{2}$ is the narrow-sense heritability, a is the additive genetic variance estimate, and e is the residual variance estimate. Standard errors for heritability estimates were calculated using the following equation,

$${SE}^{2}\left(h^{2}\right)=\left(\begin{array}{cc} \frac{h^{2}\left(1-h^{2}\right)}{a} & -\frac{h^{2}\left(h^{2}\right)}{a} \end{array}\right)\left(\begin{array}{cc} var(a) & cov(a,e)\\ cov(e,a) & var(e) \end{array}\right)\left(\begin{array}{c} \frac{h^{2}\left(1-h^{2}\right)}{a}\\ -\frac{h^{2}\left(h^{2}\right)}{a} \end{array}\right),$$

where a is the additive genetic variance and e is the residual variance. The leading row vector and following column vector are partial derivatives solved by taking the partial derivative of $h^{2}$ with respect to a and e, respectively. Components of the internal matrix were taken from the inverse of the average information matrix output by AIREMLF90. Bivariate model analyses were conducted to estimate genetic covariances between combinations of the teat traits, with which genetic correlations were calculated using the following equation,

$$r=\frac{x_{12}}{\sqrt{x_{1}x_{2}}}$$

where r is the genetic correlation, $x_{12}$ is the genetic covariance between traits 1 and 2, $x_{1}$ is the genetic variance of trait 1, and $x_{2}$ is the genetic variance of trait 2. Standard errors for genetic correlation estimates were calculated using the following equation,

$${SE}^{2}\left(r\right)=\left(\begin{array}{ccc} -\frac{r}{2x_{1}} & -\frac{r}{2x_{2}} & \frac{r}{x_{12}} \end{array}\right)\left(\begin{array}{ccc} var(x_{1}) & cov(x_{1},x_{2}) & cov(x_{1},x_{12})\\ cov(x_{2},x_{1}) & var(x_{2}) & cov(x_{2},x_{12})\\ cov(x_{12},x_{1}) & cov(x_{12},x_{2}) & var(x_{12}) \end{array}\right)\left(\begin{array}{c} -\frac{r}{2x_{1}}\\ -\frac{r}{2x_{2}}\\ \frac{r}{x_{12}} \end{array}\right)$$

where r is the genetic correlation, $x_{12}$ is the genetic covariance between traits 1 and 2, $x_{1}$ is the genetic variance of trait 1, and $x_{2}$ is the genetic variance of trait 2. Components for the matrix were also taken from the inverse of the average information matrix. Expected progeny difference (EPD) values for the sires of sows sampled were obtained from the AIREMLF90 v. 1.135 output.

## 3. Results

### 3.1. Descriptive Statistics

The number of piglets born alive was 12.8 piglets per litter.

**Figure 2:**
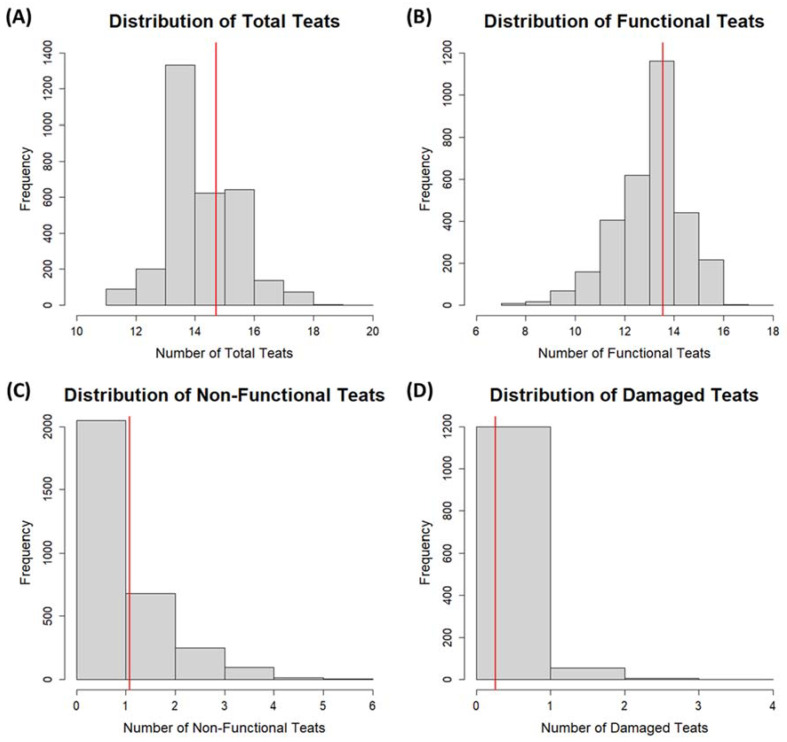
Histogram distributions of teat traits in the sample population; red line indicates the mean within each trait for Landrace × Large White F1 sows. Distributions for the total teat number, the functional teat number, and the non-functional teat number include 3099 observations (from sows at farrowing and sows at weaning) and the damaged teat number includes 1259 observations (from sows at weaning).

Distributions of the total teat number, the functional teat number, the non-functional teat number, and the damaged teat number are shown in Figure 2. Distributions for the non-functional teat number and the damaged teat number were skewed to the right.

Farm and parity means for underline traits are shown in Table 1. Across all farms, averages (±SD) of the total teat number, the functional teat number, and the non-functional teat number ranged from 14.45 ± 0.97 to 14.91 ± 1.29, 13.30 ± 1.44 to 13.96 ± 1.27, and 0.58 ± 0.94 to 1.29 ± 1.22, respectively.

Across parity groups, means (±SD) for the total teat number, the functional teat number, and the non-functional teat number ranged from 14.56 ± 1.23 to 14.85 ± 1.21, 13.43 ± 1.46 to 13.69 ± 1.37, and 0.99 ± 1.17 to 1.28 ± 1.20, respectively. For the total teat number, first parity sows had fewer (*p* < 0.05) teats when compared with all other parities (14.56 ± 1.23 vs. 14.72 ± 1.21 to 14.85 ± 1.21). First parity sows also had a lower (*p* < 0.05) functional teat number when compared with second and third parity sows (13.43 ± 1.46 vs. 13.61 ± 1.34 and 13.69 ± 1.37, respectively). Sows in parity category 6+ had a greater (*p* < 0.01) non-functional teat number than first, second, and third parity sows (1.28 ± 1.20 vs. 1.00 ± 1.13, 0.99 ± 1.17 and 1.06 ± 1.17, respectively).

### 3.2. Variance Component Estimation

Descriptive statistics and variance component estimates of underline and production traits are reported in Table 2. Heritability estimates for the total teat number and the functional teat number were of greater magnitude at farrowing relative to weaning. Yet combing the farrowing and weaning data sets produced relatively greater heritability estimates for the total teat number and the functional teat number when compared with either the farrowing or weaning data by themselves. At weaning, heritability estimates for the total teat number, the functional teat number, and the non-functional teat number were relatively greater than estimates for the damaged teat number and the number of functional mammary glands (0.15 to 0.18 vs. 0.03 to 0.06). In the farrowing dataset, the heritability estimate for the number weaned was 0.11. Yet for the weaning dataset, AIREMLF90 was unable to converge when estimating variance components for the number weaned.

Phenotypic and genetic correlation estimates among underline traits for the farrowing data and weaning data are shown in Table 3 and Table 4, respectively. Genetic correlation estimates between the total teat number and the functional teat number for the farrowing and weaning datasets were 0.78 and 0.68, respectively. Genetic correlations between the total teat number with the non-functional teat number were positive (0.22 to 0.47), while estimates between the functional teat number and the non-functional teat number were negative (−0.19 to −0.57). In the weaning dataset, the genetic correlation estimates between the total teat number, the functional teat number, and the non-functional teat number with the number of functional mammary glands were 0.17, 0.72, and −0.57, respectively. In the farrowing dataset, the genetic correlation estimates between the total teat number and the functional teat number with the number weaned were 0.25 and 0.50, respectively.

### 3.3. Linear Regression Estimates

Linear regression estimates among underline traits with the number weaned are reported in Table 5. Measured at farrowing, a one teat increase in the total teat number and the functional teat number increased (*p* < 0.05) the number weaned by 0.14 and 0.27 piglets, respectively. Measured at weaning, a one teat increase in the total teat number and the functional teat number increased (*p* < 0.05) the number weaned by 0.16 and 0.18 piglets, respectively.

Range of sire expected progeny differences for underline traits and the number of piglets weaned are shown in Table 6. For underline traits, the total teat number and the functional teat number had sire expected progeny differences ranging from −0.83 to 1.44 and −1.39 to 1.14, respectively.

## 4. Discussion

### 4.1. Descriptive Statistics

A major goal of pig production at the sow farm level is to produce piglets that survive to weaning and grow to an acceptable size, as well as sows that are able to stay in the herd and produce numerous, consistent litters. Data from the present study clearly support that a sufficient number of functional teats in lactation enhances piglet survivability and sow performance.

In the present study, 92% of teats were classified as functional, 7.3% non-functional, and 0.7% damaged. In agreement, [16] reported that the percentages of functional teats and non-functional teats were 96 and 4%, respectively. While the majority of teats are functional, the number of functional teats may be exceeded by litter size in hyper-prolific sows [17]. Therefore, enhancing the functional teat number is essential to ensuring a piglet’s ability to find a teat and suckle [18].

Means for the total teat number in the present study (14.43 and 14.93) were within the ranges reported by [5,7,9,19]. Refs. [20,21,22] reported relatively lower means for total teat numbers of 13.5 to 13.8, 12.7 to 14.4, and 13.8 to 14.1, respectively. The mean number of functional teats in the present study at farrowing (13.90) was less than the functional teat mean of 14.83 reported by [23]. Perhaps differences in teat count between populations suggest that selection for increased teat number is possible.

At weaning, the number of functional mammary glands was lower than the functional teat number, perhaps indicating that not all functional teats were being utilized by piglets during lactation. These results are supported by [24] who reported that piglets did not suckle all available functional teats the first day after birth. In the present study, it is unknown whether non-functional mammary glands at weaning entered mammary involution due to lack of nursing by the piglets, cessation of milk production, or both.

The mean damaged teat number was 0.26 teats. These teats were most likely functional before damage due to environmental influences. According to [25], sows with larger litters had more damaged teats at weaning when compared with sows with smaller litters. This suggests damage could be due to an increase in the intensity of piglet nursing behavior or perhaps increased ventral lying of the sow. The occurrence of damaged teats results from a previously functional teat becoming necrotic and then non-functional.

Besides teat functionality, sow udder morphology can influence the success of piglets in quickly finding a teat and suckling [26]. Teat distance from the abdominal midline and inter-teat distance are two important udder characteristics that can significantly influence teat use and availability. Multiparous sows, when compared with primiparous sows, have a greater mean teat pair distance, longer teat length, and larger teat diameter [18]. This perhaps explains why piglets from high parity sows take longer to first suckle and have a lower weight gain in the first 24 h postpartum than piglets from low parity sows [24].

### 4.2. Variance Component Estimation

Heritability estimates for the functional teat number (0.16 to 0.28), in the current study were similar to estimates (0.21 to 0.42) reported by [7,8,9,27]. For the non-functional teat number, heritability estimates in the current study (0.12 to 0.15) were within the wide range of previous estimates (0.02 to 0.46) reported by [7,8,9,27]. To our knowledge, previous studies have not evaluated genetic variation for the damaged teat number or the number of functional mammary glands, yet heritability estimates in the current study for these two traits were relatively low (0.03 to 0.06). By comparison, [16] reported heritability estimates for udder morphology traits of 0.11 to 0.53. Perhaps the damaged teat number is associated with udder morphology traits as certain teat characteristics may increase the likelihood that a teat is damaged by flooring materials or other environmental factors.

In the present study, heritability estimates for the total teat number and the functional teat number were greater from the combined data set when compared with the individual data sets. Perhaps this suggests collecting teat data on a large number of animals is more important than recording teat data at a certain point in time. Heritability estimates for the total teat number and the functional teat number were greater at farrowing relative to weaning. This can perhaps be explained by the presence of damaged teats at weaning increasing environmental variation or impaired ability of the technicians to classify teats at weaning. Nonetheless, results suggest evaluating the functional teat number on a large number of animals would enhance genetic parameters for functional teat count.

Genetic correlation estimates between the total teat number and the functional teat number in the current study were 0.68 to 0.78 across data sets. Similarly, Marois and Larochelle (2008) and Lundeheim et al. (2013) [7,9] reported genetic correlation estimates between the total teat number and the functional teat number of 0.83 and 0.82, respectively. Taken together, the results of these studies suggest that the total teat number and the functional teat number are different traits.

Similar to previous studies [7,9,27], the heritability estimate of the total teat number was greater than the estimated heritability of the functional teat number. Yet selection on the functional teat number may be preferred if the functional teat number enhances reproduction more than the total teat number.

Genetic correlation estimates between the total teat number and the functional teat number with the number weaned were 0.25 and 0.50, respectively. Similarly, Balzani et al. (2016) [16] estimated a favorable genetic correlation between the total teat number and a measure of piglet mortality (−0.57). In agreement, Pumfrey et al. (1980) [6] reported a positive genetic correlation between the total teat number and a measure of piglet survival (0.27). Krupa et al. (2016) [5] estimated a genetic correlation between the total teat number and the number weaned of 0.08. Collectively, these genetic correlations suggest selection for the increased total teat number or the functional teat number would enhance reproductive throughput. Yet given that the functional teat number had a more favorable association with the number weaned than the total teat number, the functional teat number would be the recommended underline trait to record and include in the breeding objective to enhance piglet survival.

The non-functional teat number had a negative genetic correlation (−0.38) with the number weaned. Similarly, Balzani et al. (2016) [16] reported a negative association between the non-functional teat number and a measure of piglet survival (−0.16). These results are not supported by [28] who reported sows with substandard teats tended to have greater piglet survival. Perhaps differences between studies can be explained by differing teat definitions. Knauer and Peppmeier (2021) [28] classified supernumerary teats while the current study and Balzani et al. (2016) [16] classified supernumerary, inverted, and damaged teats as non-functional.

Estimated genetic correlations between the functional teat number, the non-functional teat number, and the damaged teat number with the number of functional mammary glands were sizeable (0.72, −0.57 and −0.98, respectively) indicating that the number of functional mammary glands shares substantial genetic variation with these traits. These results support the idea that for a mammary gland to be functional it should have an equivalent functional teat. Hence, the number of functional mammary glands at weaning can be a useful indicator of the number of functional teats being utilized by piglets during the lactation period.

### 4.3. Linear Regression Estimates

The present study confirms substantial associations between the functional teat number and reproductive throughput. An increase of one functional teat enhanced the number of piglets weaned per sow (0.14 to 0.27). In agreement, Wiegert and Knauer (2018) [23] reported an increase of one functional teat at farrowing improved the number weaned by 0.34 piglets per litter. Similarly, [27,29] reported an increase of one functional teat enhanced litter size at weaning by 0.32 and 0.30 piglets, respectively. Taken together, these current studies support past research [20,30] demonstrating the importance of the functional teat number on reproductive throughput in swine systems.

Enhanced reproductive throughput due to a greater number of functional teats is perhaps explained by increased colostrum consumption and improved colostrum distribution. Colostrum, available for up to 24 h after a sow farrows, is needed for piglets to survive and thrive [31]. The authors of [23] reported piglet colostrum intake and sow colostrum production increased as the sow functional teat number increased. Further, [23] found variation in piglet colostrum intake was reduced as the sow functional teat number increased. In other words, a greater number of functional teats enhances colostrum distribution and improves the likelihood that a piglet receives a threshold level of colostrum needed for survival to weaning [31].

The importance of underline traits increases as litter size increases relative to functional teat count. Speckman et al. (2021) [29] divided the number of piglets born alive into quartiles: Q1 ≤ 10 piglets, Q2 = 11 to 12 piglets, Q3 = 13 to 14 piglets, and Q4 ≥ 15 piglets. The authors reported regression coefficients between functional teat count and litter size at weaning increased across quartiles (0.12, 0.27, 0.33, and 0.38, piglets, for Q1, Q2, Q3, and Q4, respectively). Hence as the ratio of initial litter size to functional teats increases, the number of functional teats becomes critical. This is supported by Vande Pol et al. (2021) [19] who cross-fostered piglets at the beginning of lactation to fewer piglets than the sow functional teat number, the same number of piglets as the functional teat number, or more piglets than the functional teat number. The authors reported lower piglet mortality when the initial litter size was less than the number of functional teats in comparison with the initial litter size being greater than the number of functional teats (7.7 vs. 17.9%).

## 5. Conclusions

The results from the current study demonstrate the importance of sow underline traits in lactation, animal well-being, and piglet throughput. The teat number, especially the functional teat number, plays a substantial role in piglet survival and the importance of the functional teat number increases when a sow has more piglets than teats available. Continued selection for larger litter sizes will be futile without the selection for traits that enhance piglet survival and litter quality, such as underline traits. Understanding the genetics behind the functional teat number in swine and including these traits in genetic selection programs will have a positive impact on sow performance and piglet survival by enhancing teat accessibility to piglets postpartum. Augmenting the ability of piglets to suckle will increase piglet colostrum intake, thus improving pre-weaning mortality rates, growth and development, and heightening passive immunity protection between the sow and piglet.

Sufficient genetic variation for the total teat number and the functional teat number during lactation was identified, signifying that improvement for teat traits is possible. Yet animal breeders should use caution when including the total teat number in the breeding goal as it had a positive correlation with the non-functional teat number. Hence, breeders should target the functional teat number. Utilizing the functional teat number in the selection index, while avoiding selecting pigs with inverted or supernumerary teats, should improve the general quality of sow teats and reproductive performance. By increasing the number of functional teats on a sow, producers can enhance the number of piglets that survive to weaning and enhance piglet well-being.

## 6. Future Direction

Measuring underline traits, at any stage of production, is labor intensive. Hence, future efforts should be made towards the automation of collecting underline trait phenotypes.

## Figures and Tables

**Table 1:** Farm and parity means for underline traits from 3099 Landrace × Large White F1 sows.

		Total Teat Number		Functional Teat Number		Non-Functional Teat Number		
		Mean	SD *	Mean	SD	Mean	SD	N
Farm	A	14.68 ^b,c^	1.20	13.30 ^a^	1.44	1.29 ^d^	1.22	669
	B	14.91 ^d^	1.29	13.62 ^b^	1.43	1.12 ^c^	1.21	660
	C	14.71 ^b,c^	1.25	13.22 ^a^	1.48	1.40 ^d^	1.28	607
	D	14.59 ^a,b^	1.18	13.69 ^b^	1.35	0.79 ^b^	0.95	486
	E	14.81 ^c,d^	1.27	13.96 ^c^	1.27	0.79 ^a,b^	1.02	458
	F	14.51 ^a,b^	1.23	13.60 ^b^	1.36	0.77 ^a,b^	1.00	124
	G	14.45 ^a^	0.97	13.78 ^b,c^	1.09	0.58 ^a^	0.94	95
Parity	1	14.56 ^a^	1.23	13.43 ^a^	1.46	1.00 ^a^	1.13	737
	2	14.72 ^b^	1.21	13.61 ^b,c^	1.34	0.99 ^a^	1.17	569
	3	14.85 ^b^	1.21	13.69 ^c^	1.37	1.06 ^a,b^	1.17	595
	4	14.72 ^b^	1.19	13.49 ^a,b^	1.52	1.14 ^b,c^	1.22	470
	5	14.77 ^b^	1.31	13.55 ^a,b,c^	1.42	1.13 ^a,b,c^	1.21	327
	6+	14.82 ^b^	1.26	13.47 ^a,b^	1.38	1.28 ^c^	1.20	401

* Standard deviation. ^a–d^ Indicates difference (*p* < 0.05) within column by farm or parity.

**Table 2:** Descriptive statistics and variance component estimates of underline and production traits for Landrace × Large White F1 sows.

	No.	Mean ± SE *	$\sigma_{P}^{2}$	$h^{2}$	SE
Farrowing traits					
Total teat number	1840	14.93 ± 0.03 ^h^	1.50	0.26	0.08
Functional teat number	1840	13.90 ± 0.09 ^e^	1.44	0.22	0.07
Non-functional teat number	1840	1.03 ± 0.03 ^a^	1.32	0.12	0.06
Weaning traits					
Total teat number	1259	14.43 ± 0.03 ^f^	1.35	0.18	0.09
Functional teat number	1259	13.02 ± 0.04 ^c^	1.99	0.16	0.10
Non-functional teat number	1259	1.15 ± 0.03 ^b^	1.18	0.15	0.08
Damaged teat number	1259	0.26 ± 0.02	0.31	0.03	0.03
Number of functional mammary glands	1259	9.67 ± 0.05	2.42	0.06	0.10
Number weaned	1259	10.56 ± 0.05	2.78	0.11	0.09
Combined					
Total teat number	3099	14.72 ± 0.02 ^g^	1.45	0.37	0.06
Functional teat number	3099	13.54 ± 0.03 ^d^	1.70	0.28	0.08
Non-functional teat number	3099	1.08 ± 0.02 ^a,b^	1.28	0.14	0.05

* Standard error. ^a–h^ Indicates difference (*p* < 0.01) between means within trait.

**Table 3:** Phenotypic (above diagonal) and genetic (below diagonal) correlation estimates ±SE among underline traits (at farrowing) and production traits for 1840 Landrace × Large White F1 sows.

Trait	Total Teat Number	Functional Teat Number	Non-Functional Teat Number	Number Weaned
Total teat number	-	0.26 (0.08)	0.11 (0.07)	0.09 (0.09)
Functional teat number	0.78 (0.05)	-	−0.04 (0.06)	0.15 (0.09)
Non-functional teat number	0.47 (0.12)	−0.19 (0.12)	-	−0.08 (0.07)
Number weaned	0.25 (0.07)	0.50 (0.08)	−0.38 (0.14)	-

**Table 4:** Phenotypic (above diagonal) and genetic (below diagonal) correlation estimates ±SE among underline traits (at weaning) for 1259 Landrace × Large White F1 sows.

Trait	TT	FT	NFT	DT	FMG
Total teat number	-	0.19 (0.10)	0.04 (0.08)	−0.01 (0.03)	0.03 (0.09)
Functional teat number	0.68 (0.06)	-	−0.14 (0.09)	−0.02 (0.04)	0.15 (0.11)
Non-functional teat number	0.22 (0.25)	−0.57 (0.26)	-	0.00 (0.03)	−0.09 (0.09)
Damaged teat number	−0.22 (0.33)	−0.30 (0.40)	0.04 (0.20)	-	−0.04 (0.03)
Number of functional mammary glands	0.17 (0.34)	0.72 (0.50)	−0.57 (0.55)	−0.98 (0.71)	-

Note: TT: total teat number; FT: functional teat number; NFT: non-functional teat number; DT: damaged teat number; FMG: number of functional mammary glands.

**Table 5:** Linear regression estimates among underline traits with the number weaned for 1840 and 1259 Landrace × Large White F1 sows at farrowing and weaning, respectively.

	Total Teat Number		Functional Teat Number	
	Farrowing	Weaning	Farrowing	Weaning
Number weaned ± SE	0.14 ± 0.03	0.16 ± 0.04	0.27 ± 0.03	0.18 ± 0.03

**Table 6:** Range of sire expected progeny differences (EPD) for underline traits and the number of piglets weaned from 3099 Landrace × Large White F1 sows.

	Trait			
	Total Teat Number	Functional Teat Number	Non-Functional Teat Number	Number Weaned
Range of sire EPDs	−0.83 to 1.44	−1.39 to 1.14	−0.57 to 0.65	−4.13 to 2.27

## Data Availability

Data sharing is not applicable to this article.
